# Qingda Granule Attenuates Angiotensin II-Induced Renal Apoptosis and Activation of the p53 Pathway

**DOI:** 10.3389/fphar.2021.770863

**Published:** 2022-02-10

**Authors:** Linzi Long, Xiuli Zhang, Ying Wen, Jiapeng Li, Lihui Wei, Ying Cheng, Huixin Liu, Jianfeng Chu, Yi Fang, Qiurong Xie, Aling Shen, Jun Peng

**Affiliations:** ^1^ Academy of Integrative Medicine, Fuzhou, China; ^2^ Fujian Key Laboratory of Integrative Medicine on Geriatrics, Fuzhou, China; ^3^ Chen Keji Academic Thought Inheritance Studio, Fuzhou, China; ^4^ Department of Physical Education, Fujian University of Traditional Chinese Medicine, Fuzhou, China

**Keywords:** Qingda granule, hypertension, renal injury, Ang II, p53 pathway

## Abstract

**Background:** Qingda granules (QDG) exhibit antihypertension and multiple-target-organ protection. However, the therapeutic potential of QDG on hypertensive renal injury remains unknown. Therefore, the main objective of the current study is to explore the effects and underlying mechanisms of QDG treatment on renal injury in angiotensin (Ang) II-infused mice.

**Methods and results:** Mice were infused with Ang II (500 ng/kg/min) or saline for 4 weeks with subcutaneously implanted osmotic pumps. After infusion, mice in the Ang II + QDG group were intragastrically administrated with QDG daily (1.145 g/kg/day), whereas the control group and Ang II group were intragastrically administrated with the same amount of double-distilled water. Blood pressure of the mice monitored using the CODA™ noninvasive blood pressure system revealed that QDG treatment significantly attenuated elevated blood pressure. Moreover, hematoxylin–eosin staining indicated that QDG treatment ameliorated Ang II-induced renal morphological changes, including glomerular sclerosis and atrophy, epithelial cell atrophy, and tubular dilatation. RNA-sequencing (RNA-seq) identified 662 differentially expressed transcripts (DETs) in renal tissues of Ang II-infused mice, which were reversed after QDG treatment. Kyoto Encyclopedia of Genes and Genomes (KEGG) analysis based on DETs in both comparisons of Ang II vs. Control and Ang II + QDG vs. Ang II identified multiple enriched pathways, including apoptosis and p53 pathways. Consistently, terminal deoxynucleotidyl transferase (TdT) dUTP nick-end labeling (TUNEL) staining and Annexin V staining revealed that QDG treatment significantly attenuated Ang II-induced cell apoptosis in renal tissues and cultured renal tubular epithelial cell lines (NRK-52E). Furthermore, western blot analysis indicated that Ang II infusion significantly upregulated the protein expression of p53, BCL2-associated X (BAX), cle-caspase-9, and cle-caspase-3, while downregulating the protein expression of BCL-2 in renal tissues, which were attenuated after QDG treatment.

**Conclusion:** Collectively, QDG treatment significantly attenuated hypertensive renal injury, partially by attenuating renal apoptosis and suppressing p53 pathways, which might be the underlying mechanisms.

## Introduction

Hypertension is a worldwide health problem with high cardiovascular morbidity and mortality (Aroner et al., 2016). It is reported that 23.2% (estimated 244.5 million) of the Chinese adult population aged ≥18 years had hypertension, and another 41.3% (estimated 435.3 million) had prehypertension according to the Chinese guideline (Wang Z. et al., 2018). Long term hypertension leads to adverse health effects, mainly by accelerating the damage of target organs, including the kidney (Du et al., 2021). The kidney plays an essential role in maintaining renal function and blood pressure and contributes to the development of hypertension, as well as being recognized as one of the primary target organs of hypertension (Ruiz-Hurtado and Ruilope, 2018). Moreover, most current therapeutic strategies still fail to prevent the development and progression of hypertensive renal damage, leading to a large number of hypertensive patients still ultimately suffering from end-stage renal disease (ESRD) (Dong et al., 2021). Therefore, it is urgent to explore novel therapeutic strategies to prevent hypertensive renal damage.

Hypertension is an independent risk factor for chronic kidney disease (CKD). Long term hypertension leads to renal damage, including tubular interstitial fibrosis, vascular sclerosis, and glomerular sclerosis (Johnson and Dipietro, 2013). Multiple contributors, including activation of the renin–angiotensin–aldosterone system (RAAS), inflammation, oxidative stress, endoplasmic reticulum stress, apoptosis, and mitochondrial dysfunction, had been reported to play an essential role in hypertensive renal damage (Linkermann et al., 2014; Padda et al., 2015; Gai et al., 2016; Miloradović et al., 2016). Among them, abnormal activation of the RAAS is an essential cascade mediating a wide variety of physiological and pathophysiological events in kidney diseases (Benigni et al., 2001; Suzuki et al., 2007; Zhou and Liu, 2016).

The “classical” RAAS pathway involves the conversion of angiotensin I (Ang I) to angiotensin II (Ang II) by the angiotensin-converting enzyme (ACE). Then, Ang II interacted with Ang II type 1 (AT1) receptors, resulting in vasoconstriction, aldosterone and vasopressin release, salt and water retention, sympathetic activation, increased oxidative stress, sodium reabsorption, cell proliferation, and vascular hypertrophy (Yanes et al., 2006; Sullivan et al., 2010). Therefore, inhibition of RAAS is an essential strategy for antihypertension. However, currently used antihypertension medicines targeting RAAS failed to prevent Ang II-induced renal damage (Toto, 2006). Therefore, there is an urgent need to further explore novel strategies for antihypertension and prevention of hypertensive renal injury. Moreover, recent studies have revealed that Ang II stimulation significantly induced renal cell apoptosis *in vitro* and *in vivo*, suggesting the importance of preventing or attenuating hypertension-induced renal cell apoptosis (Ning et al., 2011).

Although antihypertensive medicines have made considerable progress, the existing antihypertensive medicines have a ceiling effect (Ferdinand et al., 2020). Therefore, complementary therapies to prevent hypertension-induced renal injury are urgently needed. As a Chinese medicine formula, the Qingxuan Jiang Ya Decoction (QXJYD), prescribed by academician Keji Chen of China, has been used to treat hypertension for decades and exhibits antihypertension and target organ (including vascular and kidney) protection (Xiao et al., 2016; Liu et al., 2017; He et al., 2020). Qingda granule (QDG) was simplified from QXJYD and consisted of *Gastrodia elata* Blume (Tianma), *Uncaria rhynchophylla* (Miz.) Miz. ex Havil. (Gouteng), *Scutellaria baicalensis* Georgi (Huangqin), and *Nelumbo nucifera* Gaertn (Lianzixin) in a ratio of 12:10:6:5.

Our previous studies demonstrated that QDG treatment significantly attenuated the elevation of blood pressure in both SHR- and Ang II-infused mice, promoted vasorelaxation of the thoracic aortic rings, inhibited cell proliferation of vascular smooth muscle cells, and suppressed the activation of multiple signaling pathways (including MAPK, AKT, Ca^2+^/ERK, etc.) (Huang et al., 2019; Yu et al., 2020; Wu et al., 2021). Moreover, QDG treatment significantly alleviated cardiac inflammation, hypertrophy, apoptosis, and fibrosis and suppressed the activation of NF-κB, ROS/PI3K/AKT, and TGF-β1/Smad2/3 pathways (Cheng et al., 2021; Wu et al., 2020; Chen et al., 2021). In addition, QDG treatment also exhibited neuroprotective effects in both ischemic stroke mice and Ang II-infused mice with brain nerve injury (Zhang et al., 2020; Zhang et al., 2021).

However, the potential role of QDG on hypertensive renal injury remained to be further explored. Therefore, the purpose of the current study was to investigate the renal protection of QDG treatment in Ang II-infused mice or stimulated renal tubular epithelial cells, as well as to further explore its underlying mechanisms by performing multiple biological technologies. These findings may offer a new therapeutic strategy for treating hypertensive renal injury.

## Materials and Methods

### Materials

Ang II was purchased from Abcam (Cambridge, United Kingdom). Isoflurane was obtained from Ruiwode Life Technology Co., Ltd. (Shenzhen, Guangdong, China). Hematoxylin and eosin were purchased from Solarbio Technology Co., Ltd., (Beijing, China). terminal deoxynucleotidyl transferase (TdT) dUTP nick-end labeling (TUNEL) Apoptosis Detection Kit I POD was purchased from Boster Biological Technology Co., Ltd. (Wuhan, Hubei, China). Annexin V-AbFluor™ 647 Apoptosis Detection Kit (cat. no. KTA0004), Cell Counting Kit-8 (CCK8), and antibodies against glyceraldehyde-3-phosphate dehydrogenase (GAPDH) (cat. no. Abp57259) were obtained from Abbkine (Wuhan, Hubei, China). Radioimmunoprecipitation assay (RIPA) lysis buffer, primary antibody dilution buffer, and secondary antibody dilution buffer were supplied by Beyotime Biotechnology (Shanghai, China). Polyvinylidene fluoride (PVDF) membranes were obtained from Millipore (Billerica, MA, United States). Antibodies against Bcl-2 (cat. no. 12789-1-AP) and p53 (cat. no. 10442-1-AP) were purchased from ProteinTech (Rosemont, IL, United States). Antibodies against BAX (cat. no. 2772), caspase-3 (cat. no. 9662), and caspase-9 (cat. no. 9508) and secondary antibodies anti-mouse (cat. no. 7076) and anti-rabbit (cat. no. 7074) were purchased from Cell Signaling Technology (Danvers, MA, United States). Fetal bovine serum (FBS), Dulbecco’s modified Eagle’s medium (DMEM), trypsin-EDTA, and bicinchoninic acid (BCA) protein assay reagent kit were supplied by Thermo Fisher Scientific (Waltham, MA, United States).

### Preparation of QDG

QDG is composed of four herbs: *G. elata* Blume (Tianma), *U. rhynchophylla* (Miz.) Miz. ex Havil. (Gouteng), *S. baicalensis* Georgi (Huang Qin), and *N. nucifera* Gaertn (Lianzixin) in a ratio of 12:10:6:5. QDG was extracted and supplied by Tianjiang Pharmaceutical Co., Ltd. (Jiangsu, China).

For the animal experiment, QDG was prepared as in our previous studies. The concentrations of QDG were selected based on our previous study (Cheng et al., 2021). Briefly, QDG power was dissolved in double-distilled water (dd H_2_O) to the indicated concentration immediately before use. For cell culture experiments, the stocking solution of QDG was prepared in serum-free medium to 100 mg/ml and was prepared with completed medium to indicated concentrations of QDG before use.

### Animals and Experimental Protocols

Male C57BL/6 mice (28 ± 1 g, 8 weeks) were purchased from SLAC Laboratory Animal Technology Co., Ltd. (Shanghai, China). All mice were raised under specific pathogen-free conditions, with controlled humidity (50%–60%) and temperature (23 ± 1°C), 12 h of light/dark cycle, and free access to food and water. After 1 week of adaptation, the mice were randomly divided into three groups (*n* = 5 each): Control, Ang II, and Ang II + QDG groups. Mice in the Ang II or Ang II + QDG group were infused with Ang II (500 ng/kg/min), while mice in the Control group were infused with saline for 4 weeks by subcutaneously implanting micro osmotic pumps. Mice in the Ang II + QDG group were intragastrically administrated with QDG daily (1.145 g/kg/day; 200 μl for each mouse), whereas mice in both Control and Ang II groups were intragastrically administrated with equal volumes of dd H_2_O for 4 weeks. The animal experimental protocols of this study were approved by the Animal Care and Use Committee of Fujian University of Traditional Chinese Medicine and were carried out in accordance with the National Institutes of Health Guidelines for the Care and Use of Laboratory Animals.

### Blood Pressure Measurement

The blood pressure, including systolic blood pressure (SBP), diastolic blood pressure (DBP), and mean arterial pressure (MAP), was measured prior to the experiment and once a week for 4 weeks by using the tail-cuff plethysmograph method with the CODA™ noninvasive blood pressure system (Kent Scientific, Torrington, CT, United States).

### Histological Analysis

Hematoxylin–eosin (HE) staining was performed to evaluate the morphological changes of renal tissues from each group. Briefly, renal tissues were fixed with 4% paraformaldehyde solution for 48 h, then embedded in paraffin, cut into 4 µm sections, and stained with hematoxylin for 1 min and eosin for 2 s. Then, the sections were visualized under an optical microscope (Leica, Wetzlar, Germany) at a magnification of ×400.

### RNA Sequencing (RNA-seq)

The renal tissues were stored in RNA later (Takara, Beijing, China) at room temperature for 1 h and moved to −20°C for long-time storage. Total RNA was extracted by a TRIzol reagent (Thermo Fisher Scientific, Waltham, MA, United States) according to the manufacturer’s protocol. The concentration and quality of total RNA were measured by both Qubit 3.0 and Agilent 2100 Bioanalyzer. RNA samples with a RIN value of 7 or higher were used for further experiments.

RNA-seq was performed at Capital Bio Technology (Beijing, China). In brief, rRNA was removed from total RNA by a NEB Next rRNA Depletion Kit (NEB, Ipswich, MA, United States) according to the instructions. rRNA-depleted total RNA was fragmented, and the poly(A)-tailed mRNA molecules was generated by a NEB Next Ploy(A) mRNA Magnetic Isolation Module Kit (NEB, Ipswich, MA, United States) according to the manufacturer’s instructions. Moreover, the final libraries were quantified on an Agilent 2100 Bioanalyzer using a KAPA Library Quantification Kit (KAPA Biosystem, Wilmington, MA, United States) and subjected to paired-end sequencing on an Illumina HiSeq sequencer (Illumina, San Diego, CA, United States).

The raw data were processed as described previously (Wu et al., 2021). The selected genes were further analyzed in the context of the information obtained from the database of Gene Ontology (GO) and Kyoto Encyclopedia of Genes and Genomes (KEGG).

### Cell Culture and QDG Treatment

The renal tubular epithelial cell line NRK-52E was purchased from BeNa (Beijing, China) and cultured in DMEM containing 10% FBS, 100 units/ml penicillin, and 100 mg/ml streptomycin. The cell lines were cultured in a humid environment at 37°C and 5% CO_2_. The NRK-52E cells were cultured in six-well plates at a density of 0.8 × 10^5^ cells per well. After being cultured for 24 h, NRK-52E cells were incubated in minimum medium (0.5% FBS) for around 8 h and were divided into three groups: Control group, Ang II group, and Ang II + QDG group. Then, cells in Ang II and Ang II + QDG groups were incubated with Ang II (1 μM), followed by treatment with QDG (25 and 50 μg/ml) in the Ang II + QDG group for 48 h.

### TUNEL Staining

To assess cell apoptosis in renal tissues from each group, renal tissues were fixed in 4% paraformaldehyde, embedded in paraffin, sectioned at 4 μm, and stained using a TUNEL kit according to the manufacturer’s instructions. The specific experimental procedures for TUNEL assay were performed as previously described (Cheng et al., 2021). Slides were visualized under an optical microscope (Leica, Wetzlar, Germany) at a magnification of ×400, and five fields of view were randomly selected for each slide. Positively stained cells in each field were determined using ImageJ software.

For NRK-52E cells with or without Ang II stimulation and QDG treatment, the cells were fixed in 4% paraformaldehyde and incubated for 30 min at room temperature. The next step was the same as described above. Cells with a dark-brown nucleus were defined as apoptotic cells, while cells without or with a light-brown nucleus were defined as normal (non-apoptotic cells). The percentage of apoptotic cells was calculated as the ratio of the number of TUNEL-positive cells to the total number of cells examined.

### Cell Viability Analysis

The cell viability of NRK-52E cells was determined by CCK8. Briefly, the cells were seeded into 96-well plates (2 × 10^3^ per well) containing complete culture medium and cultured at 37°C in 5% CO_2_ overnight. The indicated concentrations of QDG were added to each well for 48 h. At the end of treatment, a CCK8 reagent (10 µl per well) was added to each well and incubated at 37°C for 2 h in the dark. The absorbance was determined by an enzyme-labeling instrument (Multiskan FC, Thermo Fisher Scientific) at 450 nm wavelength. The cell viability of untreated cells was set as 100%.

### Annexin V Staining

To provide a comparative assay of apoptosis by Annexin V staining, the NRK-52E cells were seeded into six-well plates at a density of 0.8 × 10^5^ cells per well. After being cultured for 24 h, NRK-52E cells were cultured in serum-free DMEM for 12 h and then were stimulated with Ang II (1 μM) and treated with or without QDG (25 μg/ml or 50 μg/ml) in 0.5% of minimum medium (0.5% FBS) for a total of 24 h. At the end of treatment, cells were harvested and stained with Annexin V and propidium iodide (PI) according to the manufacturer’s protocol. Cell apoptosis was detected and quantified by FACS (fluorescence-activated cell sorting) analysis.

### Western Blot Analysis

The cell lysis buffer with protease inhibitor and PMSF was added to the renal tissues, which were then ground with a low-temperature grinder, applied on ice for 30 min, and centrifuged at 4°C and 14,000 *g* for 15 min. The protein concentration was quantified by BCA assay. A total of 50 μg protein from each sample was separated on 10% SDS-PAGE gel, transferred to PVDF membranes, and blocked with 0.5% bovine serum albumin for 2 h followed by incubation with the primary antibodies (overnight at 4°C), including rabbit anti-BAX (1:1,000), rabbit anti-BCL2 (1:1,000), rabbit anti-p53 (1:1,000), rabbit anti-cle-caspase-3 (1:1,000), rabbit anti-GAPDH (1:5,000), and mouse anti-cle-caspase-9 (1:1,000). Then, the membrane was washed with TBST and then incubated with secondary antibody at room temperature for 1 h. At the end of incubation, the membranes were washed with TBST, and then detection was performed using an electrochemiluminescence (ECL) kit. The expression of GAPDH protein was used as an internal control. The protein levels were analyzed with ImageJ software.

### Statistical Analysis

The data were presented as mean ± SD. All statistical analyses were performed using the SPSS software (Version 22.0) for Windows (SPSS, Inc., Chicago, IL, United States). One-way analysis of variance was used to compare differences among three groups, when conforming to a normal distribution. The nonparametric Kruskal–Wallis test was used to compare differences among the three groups when not consistent with the normal distribution. A *p*-value <0.05 was considered as statistically significant.

## Results

### QDG Attenuates Ang II-Induced Elevation of Blood Pressure and Renal Injury

**FIGURE 1:**
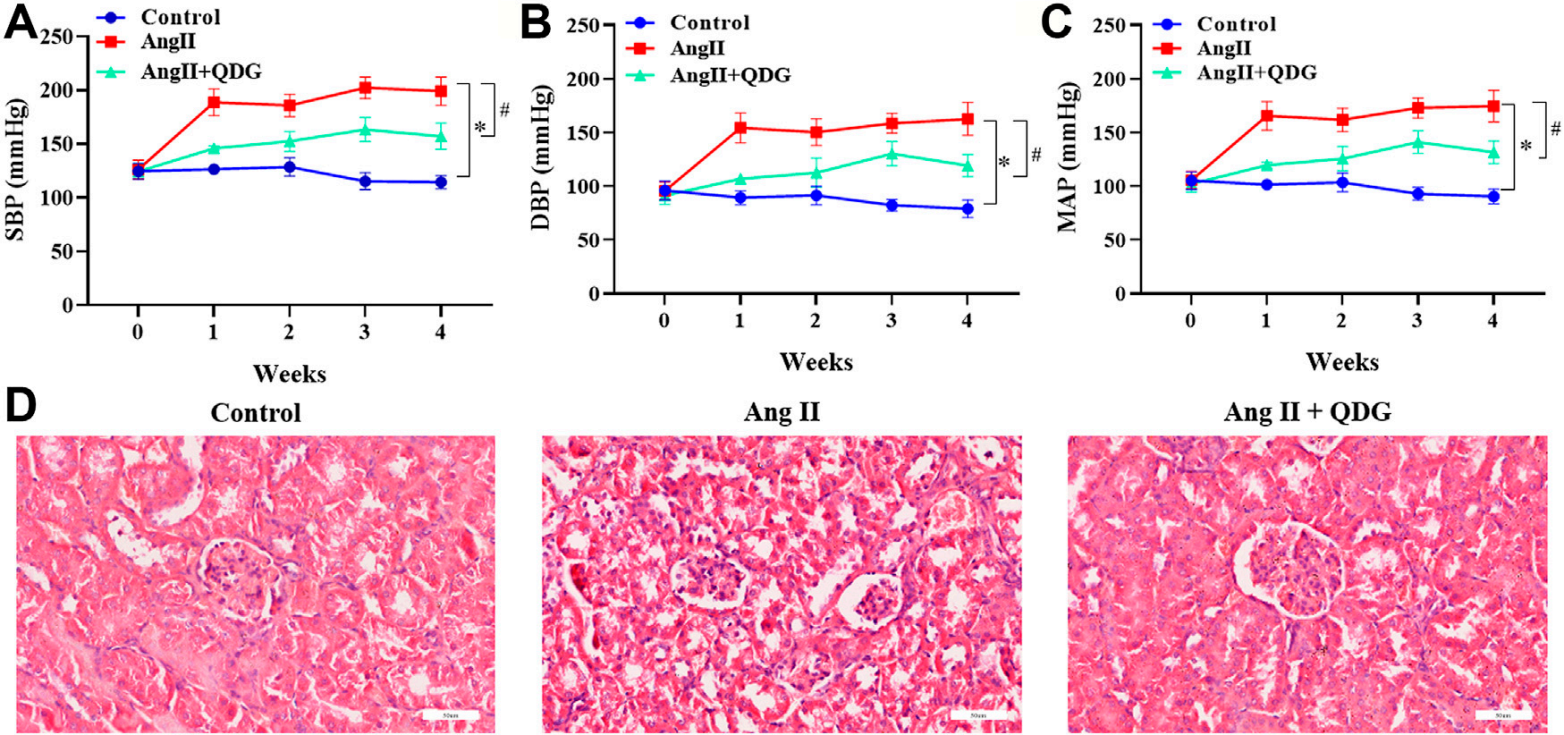
QDG attenuates Ang II-induced elevation of blood pressure and renal injury. **(A)** SBP, **(B)** DBP, and **(C)** MAP of mice from each group were monitored prior to the experiment and once a week for 4 weeks by using the CODA™ noninvasive blood pressure system. **(D)** HE staining was performed to determine the pathological changes of renal tissues from each group. The representative images were taken at a magnification of ×400 (scale bar 60 μm). *n* = 5 for each group. Data were presented as mean ± SD; *p < 0.05 vs. the Control group, #*p* < 0.05 vs. the Ang II group.

The effect of QDG on hypertension-induced renal injury was assessed using Ang II-infused mice. Consistent with our previous studies (Na et al.; Zhang et al., 2020; Cheng et al., 2021; Wu et al., 2021), Ang II infusion significantly increased the blood pressure, including SBP (Figure 1A; *p < 0.05, vs. the Control group), DBP (Figure 1B; *p < 0.05, vs. the Control group), and MAP (Figure 1C; *p < 0.05, vs. the Control group) of mice, which were attenuated after QDG treatment. Moreover, HE staining revealed significant renal pathological changes, including glomerular sclerosis and atrophy, epithelial cell atrophy, and tubular dilatation, which were alleviated after QDG treatment (Figure 1D). These results suggest that QDG treatment might relieve Ang II-induced hypertension and renal injury in Ang II-infused mice.

### Identification of QDG Treatment on DETs in Renal Tissues of Ang II-Infused Mice

**FIGURE 2:**
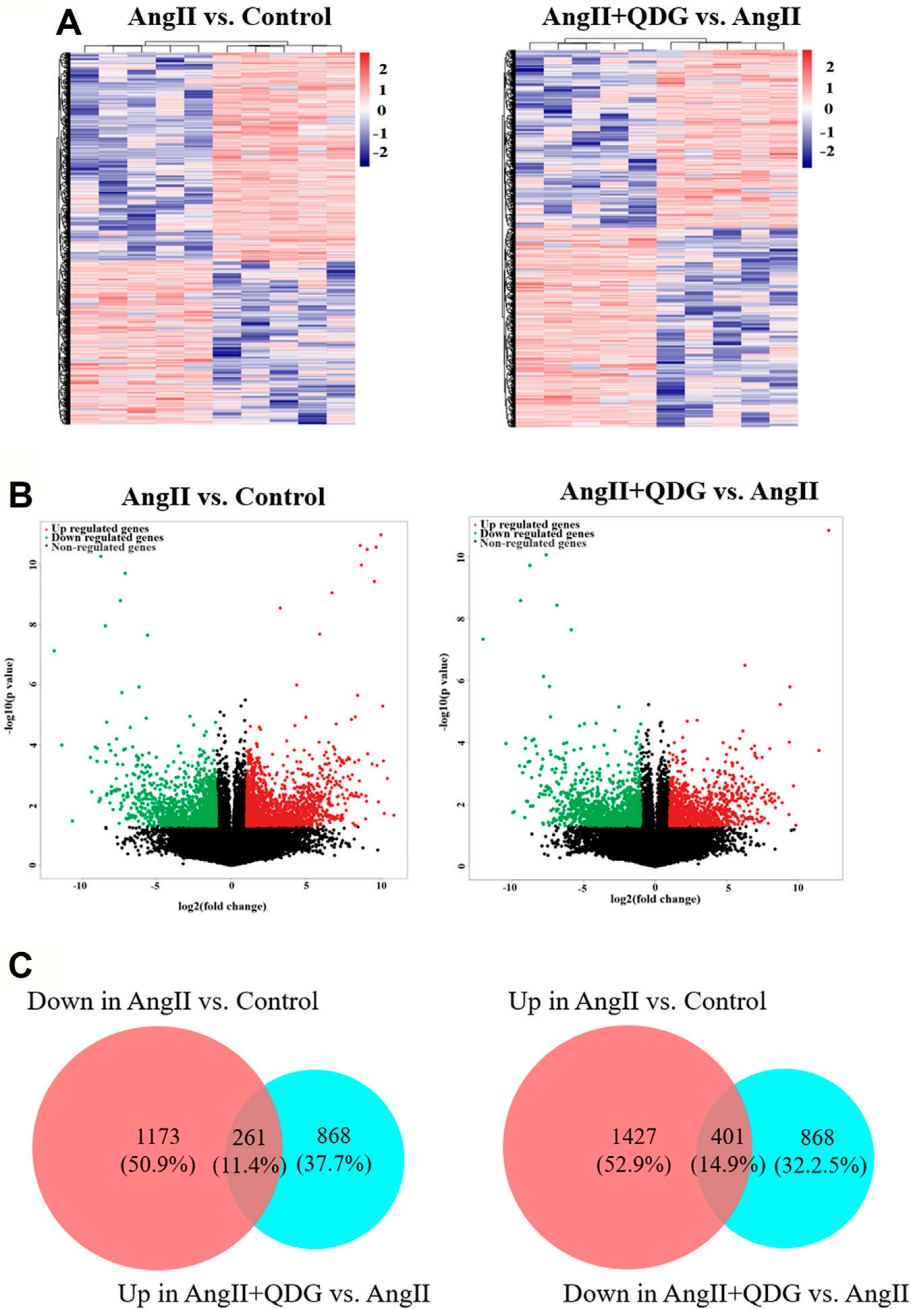
QDG treatment on gene expression profiling of renal tissues in Ang II-infused mice. RNA-seq was performed to determine the DETs in renal tissues from each group. **(A)** Hierarchical clustering plots and **(B)** volcano plots were used to compare gene expression profiles (|fold change| ≥ 2, *p* < 0.05). **(C)** Integrative analysis was performed to identify the integrated transcripts between the two comparisons. The overlapped area in the left panel represents decreased transcript number (261) in the Ang II group but increased in the Ang II + QDG group. The overlapped area in the right panel represents increased transcript number (401) in the Ang II group but decreased in the Ang II + QDG group.

To explore the underlying mechanisms of QDG on hypertensive renal protection, RNA-seq was performed to identify the differentially expressed transcripts (DETs) in renal tissues among different groups (GSE182517; https://www.ncbi.nlm.nih.gov/geo/query/acc.cgi?acc=GSE182517). As shown in Figures 2A,B (left panels), Ang II infusion significantly upregulated the expression of 1,427 transcripts and downregulated the expression of 1,173 transcripts in renal tissues of mice, compared to the Control group, while QDG treatment significantly upregulated the expression of 868 transcripts and downregulated the expression of 868 transcripts, compared with the Ang II group (Figures 2A,B, right panel). The integrative analysis between the two comparisons (Ang II vs. Control and Ang II + QDG vs. Ang II) identified 261 downregulated transcripts (Figure 2C, left panel) and 401 upregulated transcripts (Figure 2D, right panel) in the Ang II group, which were reversed after QDG treatment.

### Enrichment of Biological Processes and Signaling Pathways After QDG Treatment in Renal Tissues of Ang II-Infused Mice

**FIGURE 3:**
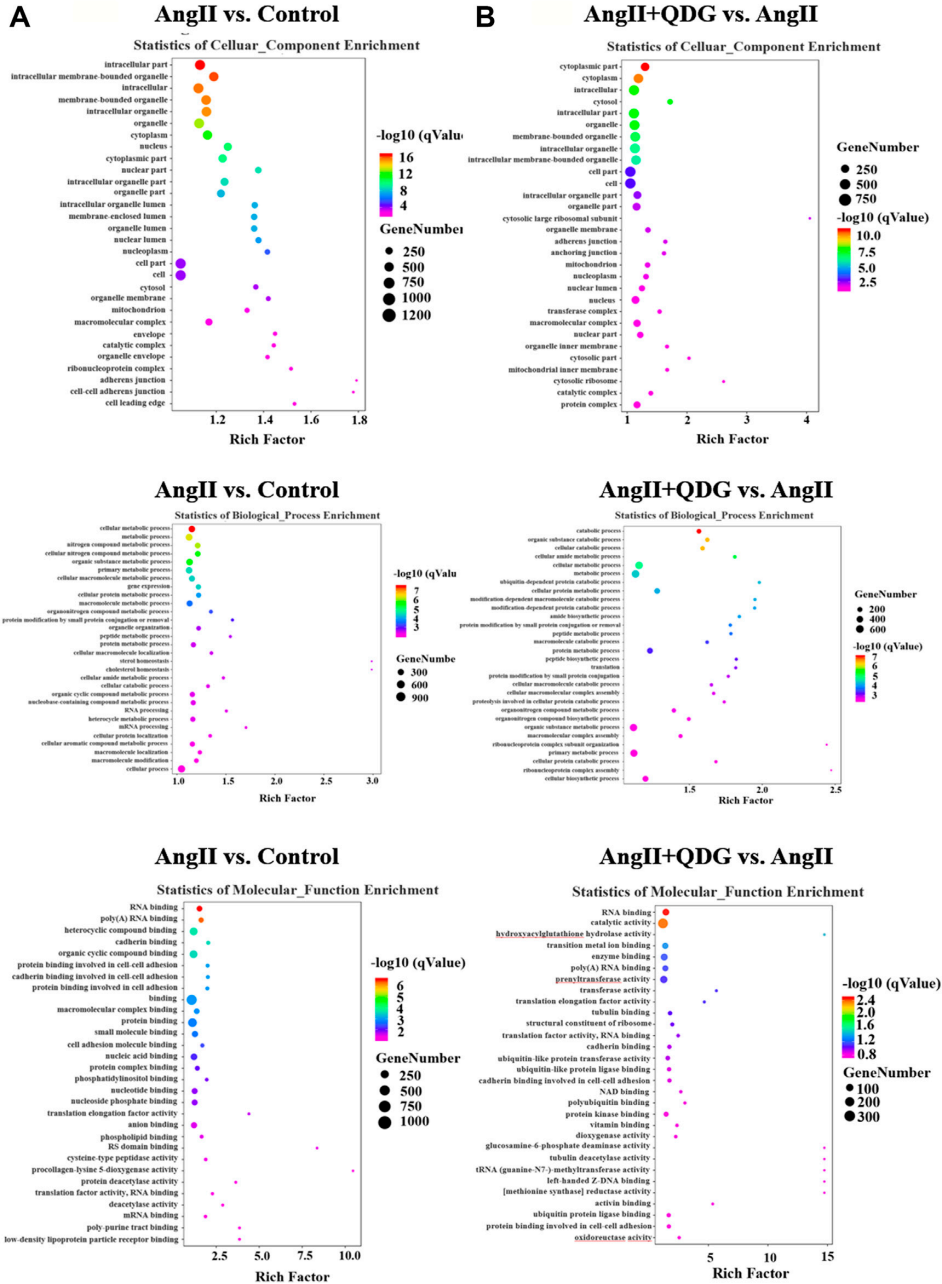
GO enrichment analysis. GO enrichment analysis was performed based on the DETs from both comparisons of **(A)** Ang II vs. Control and **(B)** Ang II + QDG vs. Ang II. The top 30 enriched items of the cellular composition (left panel), biological processes (middle panel), and molecular function (right panel).

GO enrichment analysis based on the DETs between Ang II and Control groups revealed multiple predicted potential functions. As shown in Figure 3A, cellular component (CC) analysis indicated that DETs were mainly involved in the intracellular part, intracellular membrane bounded organelle, and mitochondrion. Biological process (BP) analysis showed that DETs were mainly associated with metabolism, gene expression, and organelle organization. In the molecular function (MF) category, DETs were significantly enriched in binding, translation factor activity, etc. Moreover, GO enrichment analysis based on the DETs between Ang II + QDG and Ang II groups revealed multiple predicted potential functions. As shown in Figure 3B, CC analysis showed that DETs were associated with the cytoplasmic part, intracellular part, and cell part. As to BP, DETs were significantly enriched in the catabolic process, amide biosynthesis, and translation. The MF analysis for these DETs includes binding, catalytic activity, and translation elongation factor activity.

**FIGURE 4:**
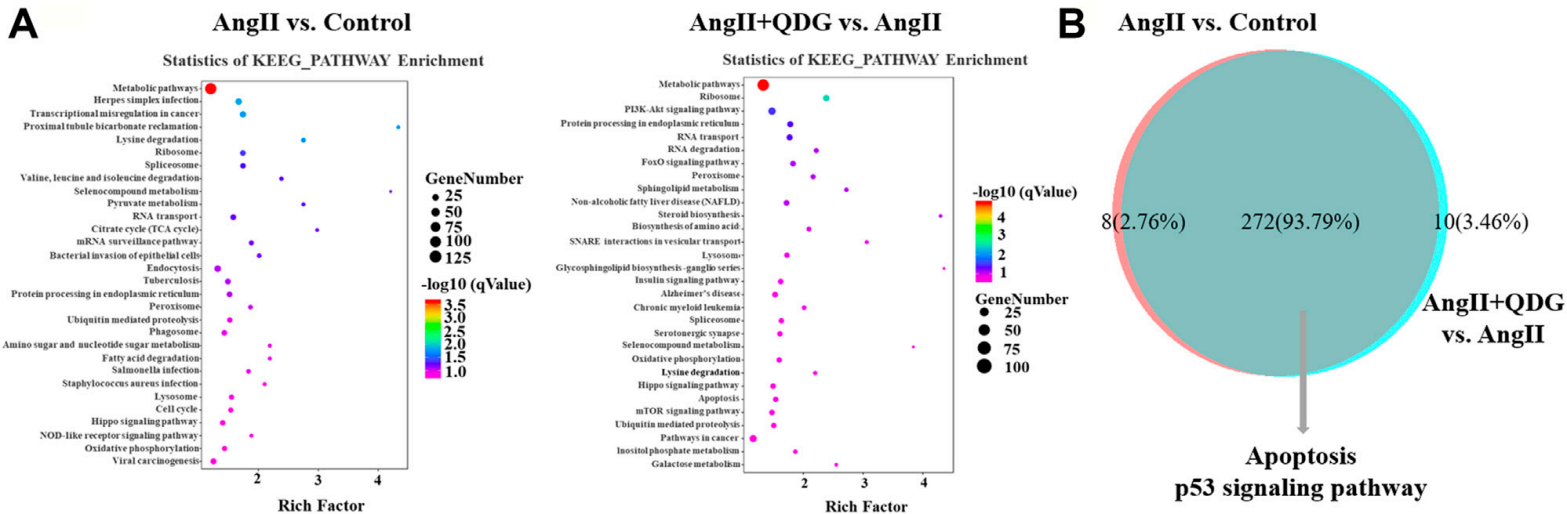
KEGG pathway enrichment analysis. **(A)** KEGG enrichment analysis was performed to identify the enriched signaling pathway in both comparisons of Ang II vs. Control (left panel) and Ang II + QDG vs. Ang II (right panel). The top 30 enriched signaling pathways were presented. **(B)** Integrative analysis was performed to identify the integrated transcripts between the two comparisons. The overlapped area represents the number of enriched signaling pathways in both comparisons of Ang II vs. Control and Ang II + QDG vs. Ang II.

KEGG pathway analysis based on the DETs between Ang II and Control groups revealed multiple enriched signaling pathways, including apoptosis, p53 signaling pathway, and MAPK signaling pathway (Figure 4A). Moreover, KEGG pathway analysis based on the DETs between Ang II + QDG and Ang II groups demonstrated multiple enriched signaling pathways, including oxidative phosphorylation, apoptosis, and p53 signaling pathways (Figure 4A). The integrative analysis between the two comparisons (Ang II vs. Control and Ang II + QDG vs. Ang II) demonstrated that multiple signaling pathways were enriched in both comparisons (Figure 4B). Notably, we find that both cell apoptosis and p53 signaling pathways were significantly enriched in both comparisons (Figure 4C), which encouraged us to further explore the regulatory effects of QDG on cell apoptosis and activation of the p53 signaling pathway in renal tissues of Ang II-infused mice.

### QDG Attenuates Ang II-Induced Renal Cell Apoptosis *In Vivo* and *In Vitro*

**FIGURE 5:**
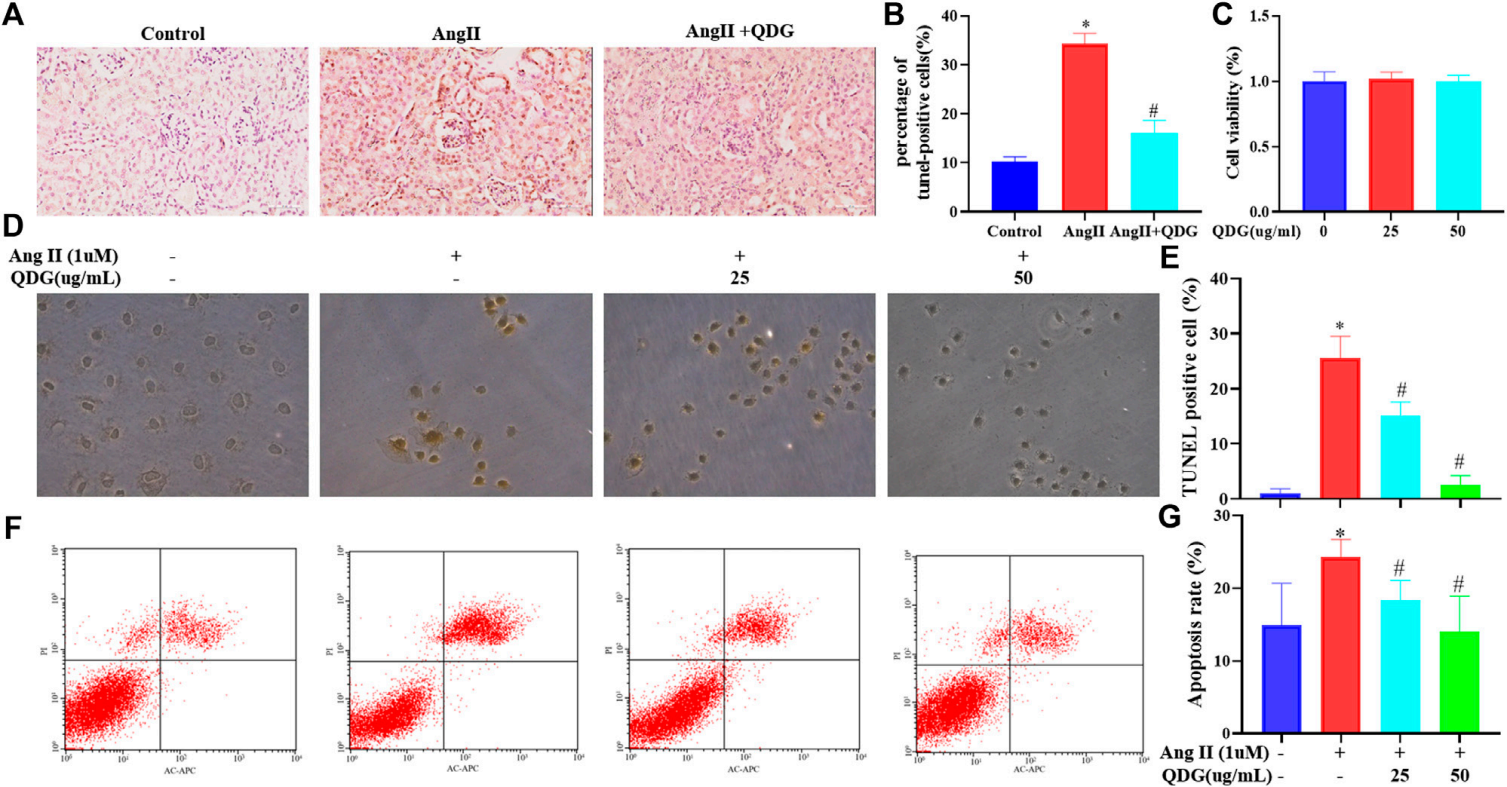
QDG attenuates Ang II-induced renal cell apoptosis *in vivo* and *in vitro*. The TUNEL assay was performed to observe cell apoptosis in renal tissues of mice from each group **(A)** and the percentage of TUNEL-positive cells **(B)**. Viability of NRK-52E cells after treatment with QDG, as analyzed by the CCK-8 assay **(C)**. The TUNEL assay was performed to observe cell apoptosis in NRK-52E cells from each group **(E)** and the percentage of TUNEL-positive cells **(D)**. Representative dot plot of Annexin V- and PI-stained cells **(F)** and a columnar graph that comprised the percentage of apoptosis in NRK-52E cells **(G)**. Data were presented as mean ± SD; *p < 0.05 vs. the Control group, #*p* < 0.05 vs. the Ang II group.

Further verification of QDG treatment on cell apoptosis of renal tissues by performing TUNEL staining revealed an increase of the percentage positively TUNEL-stained cells in renal tissues of Ang II-infused mice (Figures 5A,B; *p < 0.05 vs. the Control group), while QDG treatment significantly reduced the percentage of positively TUNEL-stained cells (Figures 5A,B; #*p* < 0.05, vs. the Ang II group), confirming the effect of QDG on attenuation cell apoptosis in renal tissues in Ang II-infused mice. We further assessed the effect of QDG treatment on cell apoptosis of a rat renal tubular epithelial cell line (NRK-52E) by performing TUNEL assay and found that 25 and 50 μg/ml of QDG treatment did not affect the cell viability of NRK-52E (Figure 5C) but attenuated the Ang II stimulation-induced cell apoptosis of NRK-52E cells (Figures 5D,E; *p < 0.05, vs. the Control group, #*p* < 0.05, vs. the Ang II group). Consistently, by performing Annexin V/PI staining, we found that Ang II stimulation significantly increased the percentage of cell apoptosis, which was attenuated after QDG treatment (Figures 5F,G; *p < 0.05, vs. the Control group, #*p* < 0.05, vs. the Ang II group).

### QDG Activates the p53 Signaling Pathway *In Vivo*

**FIGURE 6:**
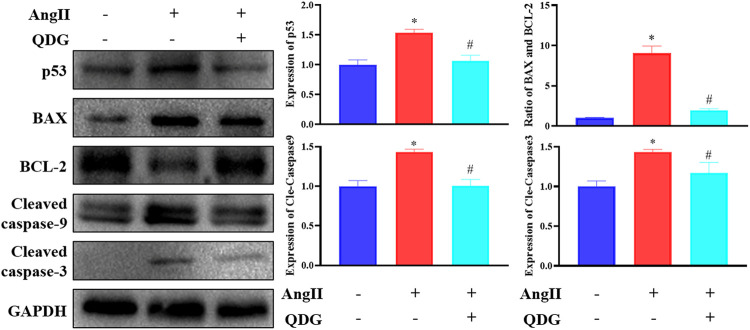
QDG inhibits activation of p53 signaling pathways induced by angiotensin (Ang II). Western blot analysis was performed to determine the protein expression of p53, BAX, BCL2, and cle-caspase-9/caspase-3. GAPDH was used as the internal control. Data were presented as mean ± SD; *p < 0.05 vs. the Control group, #*p* < 0.05 vs. the Ang II group.

To further verify the regulatory effect of QDG treatment on the activation of the p53 pathway, western blot confirmed that Ang II infusion significantly upregulated the protein expression of p53, which was downregulated after QDG treatment. Moreover, by determination of the expression apoptosis-related proteins using western blot analysis, it was revealed that Ang II infusion significantly upregulated the expression of proapoptotic proteins Bax, cle-caspase-9 and cle-caspase-3 but downregulated the expression of antiapoptotic protein Bcl-2 (Figure 6; *p < 0.05, vs. the Control group), which were all reversed after QDG treatment (Figure 6; #*p* < 0.05, vs. the Ang II group).

## Discussion

Long-term hypertension leads to multiple target organ damage, including renal damage. Our previous studies demonstrated that QDG exhibits antihypertension and multiple-target-organ protection (Huang et al., 2019; Wu et al., 2020; Yu et al., 2020; Zhang et al., 2020; Chen et al., 2021; Cheng et al., 2021; Wu et al., 2021). However, the therapeutic effects of QDG on hypertensive renal injury remained to be further explored. For the first time, the current study revealed that QDG treatment not only attenuated the elevated blood pressure but also alleviated renal cell apoptosis and mediated multiple signaling pathways (including the p53 pathway) in Ang II-infused mice. These results highlighted that QDG exhibited antihypertension and antiapoptotic effects on hypertensive renal tissues, and mediation by the p53 signaling pathway might be one of the underlying mechanisms.

Despite the development and combination of antihypertension medicines, a large number of hypertensive patients are still ultimately suffering from ESRD (Dong et al., 2021). Therefore, it is urgently required to explore and develop effective strategies for treatment of hypertension and hypertensive renal injury. As a Chinese medicine formula simplified from QXJYD, QDG exhibits antihypertension and vascular and cardiac protection, as well as neuroprotection (Huang et al., 2019; Wu et al., 2020; Yu et al., 2020; Zhang et al., 2020; Chen et al., 2021; Cheng et al., 2021; Wu et al., 2021). In addition, our current study revealed that QDG treatment not only attenuated the elevation of blood pressure but also significantly reduced Ang II-induced renal pathological changes, suggesting that QDG might be used as a complementary therapy to attenuate hypertension and prevent hypertensive target organ injury (including renal injury). However, renal damage should further be confirmed by determination of proteinuria expression or in other hypertensive animal models. Moreover, whether the renal protection of QDG treatment is dependent on blood pressure or not should be further addressed in future studies.

Due to the complicated mechanisms of Ang II-induced renal injury and QDG treatment in attenuating renal injury, we therefore performed RNA-seq, GO, and KEGG enrichment analyses to further explore their complicated mechanisms. Our current study identified 401 upregulated and 261 downregulated transcripts in renal tissues of mice in the Ang II group, which were reversed after QDG treatment. Among these DETs, upregulation of NOX4 (Kumar et al., 2020) and downregulation of ANGPT1 (Loganathan et al., 2018) had been reported to play an essential role in renal injury, which might be the targets of QDG on attenuation of hypertensive renal injury. Therefore, our current study suggests multiple targets of QDG treatment on hypertensive renal injury. However, further validation of QDG targets should be performed in future studies.

Abnormal activation of multiple signaling pathways, including TGF-β1/Smad and NF-κB signaling pathways, had been reported to play an essential role in hypertensive renal injury (Liu et al., 2017; Yan et al., 2018). Based on the DETs in the comparisons of both Ang II vs. Control and Ang II + QDG vs. Ang II, GO analysis enriched multiple biological processes, including translation, translation factor activity, catalytic activity of cell parts, and mitochondria, which might be involved in hypertensive renal injury. Moreover, KEGG enrichment analysis demonstrated that multiple signaling pathways (including MAPK and PI3K-Akt signaling pathways) were significantly enriched in both comparisons. Notably, integrative analysis between two comparisons found that both apoptosis and p53 signaling pathways were enriched. However, the regulatory effects of QDG on renal cell apoptosis and the p53 signaling pathway need to be further investigated.

As one of the characteristics of hypertensive renal injury, renal tubular epithelial cell (RTEC) apoptosis leads to the progression of renal tubulointerstitial fibrosis (Docherty et al., 2006; Ning et al., 2011; Johnson and Dipietro, 2013; Schelling, 2016; Russa et al., 2017; Dan et al., 2018), contributing to the development of end-stage nephropathy. Consistent with a previous study (Gu et al., 2021), the current study revealed that Ang II infusion significantly increased the percentage of cell apoptosis in renal tissues and cultured RTEC, which were attenuated after QDG treatment, suggesting the potential of QDG treatment for both antihypertension and attenuation of renal injury. However, the role of QDG treatment on renal inflammation, oxidative stress, and fibrosis should be further explored in future studies.

As one of the key regulators of apoptosis, p53 can be activated and stabilized through post-translational modification pathways, including ubiquitination, phosphorylation, and acetylation (Kruiswijk et al., 2015). Numerous studies have supported the involvement of p53 in cell apoptosis in multiple renal diseases (Tang et al., 2018; Wang Y. et al., 2018; Liu et al., 2019). Overexpression of p53 increases the expression of apoptotic protein Bax and decreases the expression of antiapoptotic protein Bcl-2, leading to cell apoptosis (Liu and Dan, 2018). Ang II induced upregulation of BAX and downregulation of Bcl-2 in NRK-52E cells (Ning et al., 2011). Consistently, our current study found that the protein expression of p53, Bax, cle-caspase-9 and cle-caspase-3 was significantly upregulated and that of Bcl-2 was downregulated, while being partly reversed after QDG treatment in renal tissues of mice. These studies suggest that suppression of Ang II-induced p53 signaling pathway activation might be one of the underlying mechanisms of QDG attenuation of hypertensive renal cell apoptosis. In future studies, we intend to perform a series of experiments including using p53 transgenic mice or p53 inhibitors to definitively confirm the antiapoptotic role of QDG through the p53 pathway. In addition, the regulatory effect of QDG on other enriched signaling pathways and DETs should be further addressed in future studies.

## Conclusion

In summary, the current study proved that QDG treatment significantly attenuated renal cell apoptosis in Ang II-infused mice model or Ang II-stimulated NRK-52E cells, and suppressing the activation of p53 pathway might be one of the underlying mechanisms, which may offer a new therapeutic strategy for treating hypertensive renal injury.

## Data Availability

The RNA sequencing data used in present study are deposited at NCBI Gene Expression Omnibus (GEO) under the accession numbers GSE182517; https://www.ncbi.nlm.nih.gov/geo/query/acc.cgi?acc=GSE182517.

## Glossary

Ang II: angiotensin II
BAX: BCL2-associated X
BCA: bicinchoninic acid
BP: biological process
CC: cellular component
CCK8: Cell Counting Kit-8
CKD: chronic kidney disease
DBP: diastolic blood pressure
dd H_2_O: double-distilled water
DETs: differentially expressed transcripts
DMEM: Dulbecco’s modified Eagle’s medium
ECL: electrochemiluminescence
ESRD: end-stage renal disease
FBS: fetal bovine serum
GAPDH: glyceraldehyde-3-phosphate dehydrogenase
GO: Gene Ontology
HE: hematoxylin–eosin
KEGG: Kyoto Encyclopedia of Genes and Genomes
MAP: mean arterial pressure
MF: molecular function
PVDF: polyvinylidene fluoride
QDG: qingda granule
QXJYD: Qingxuan Jiang Ya Decoction
TUNEL: terminal deoxynucleotidyl transferase (TdT) dUTP nick-end labeling
RAAS: renin–angiotensin–aldosterone system
RIPA: radioimmunoprecipitation assay
RNA-seq: RNA sequencing
RTEC: renal tubular epithelial cells
SBP: systolic blood pressure
