# Supplementary material for: Qingda Granule Attenuates Angiotensin II-Induced Renal Apoptosis and Activation of the p53 Pathway
Source: Front Pharmacol. 2022 Feb 10;12:770863. doi: 10.3389/fphar.2021.770863 (PMC8867011; doi:10.3389/fphar.2021.770863)

Bax

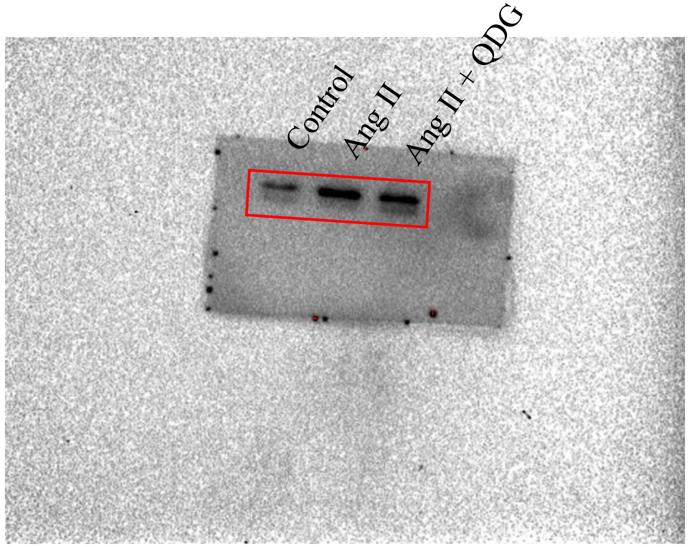
Control
Ang II
Ang II + QDG

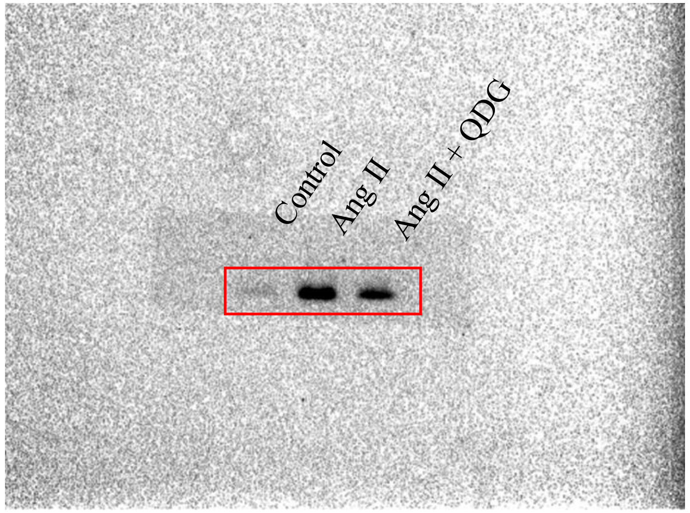
Control
Ang II
Ang II + QDG

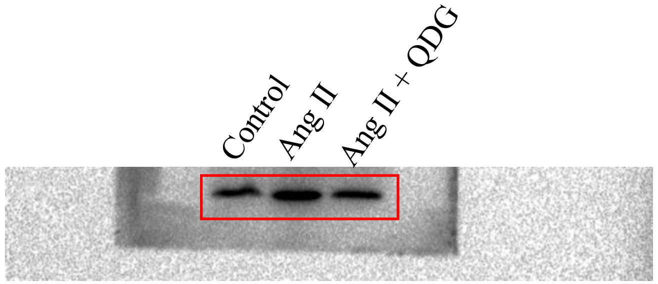
Control
Ang II
Ang II + QDG

Bcl2

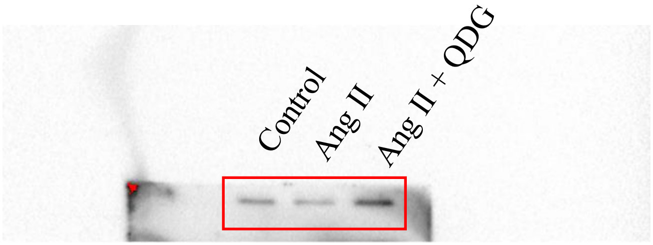
Control
Ang II
Ang II + QDG

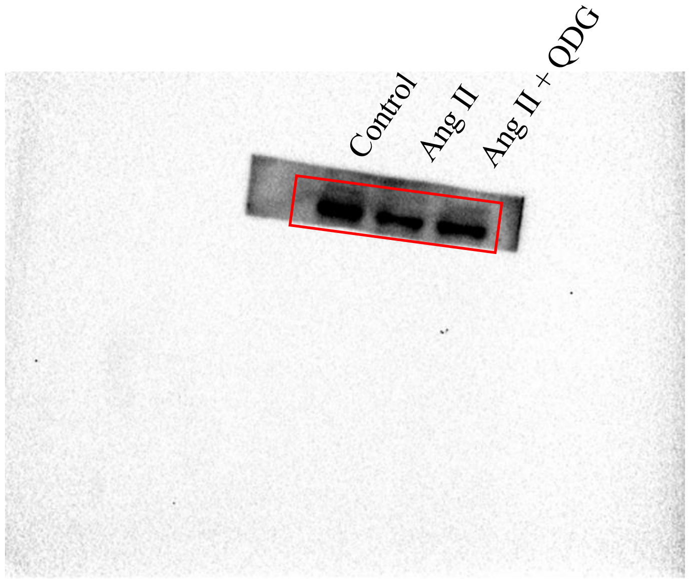
Control
Ang II
Ang II + QDG

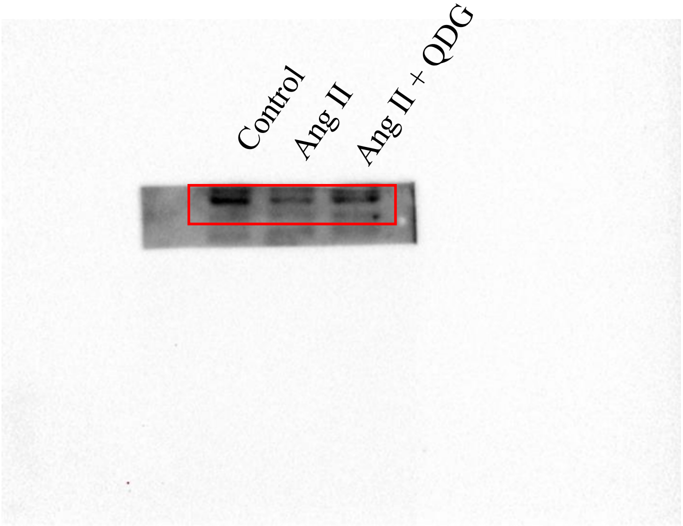
Control
Ang II
Ang II + QDG

Cleaved caspase-9

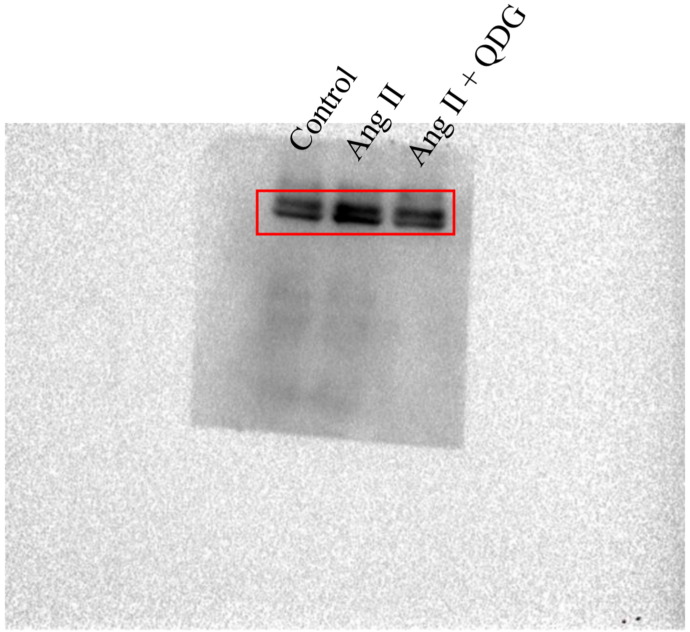

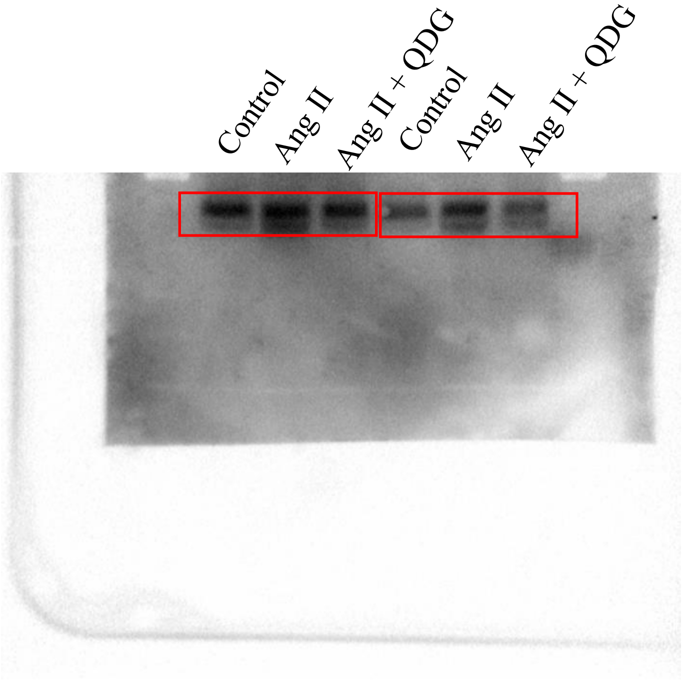

Cleaved caspase-3

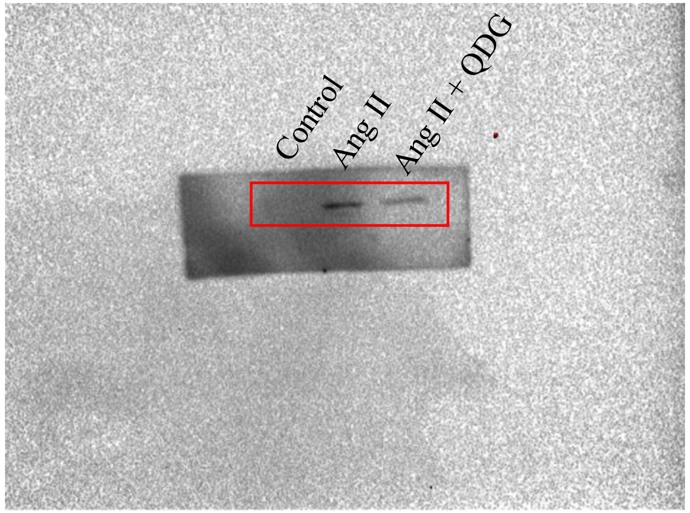

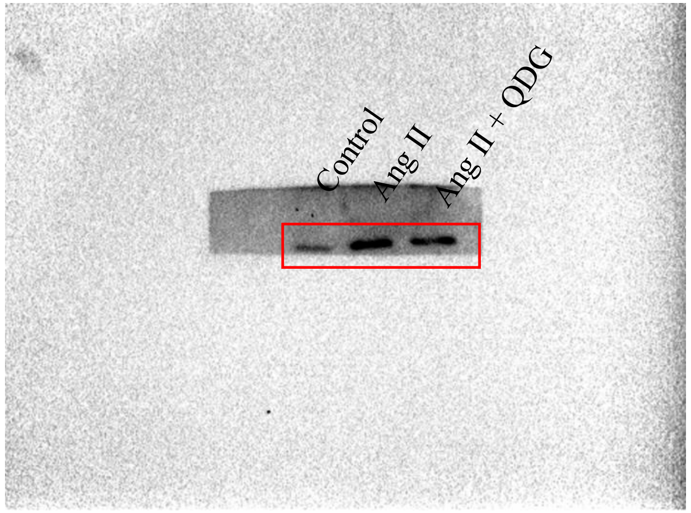

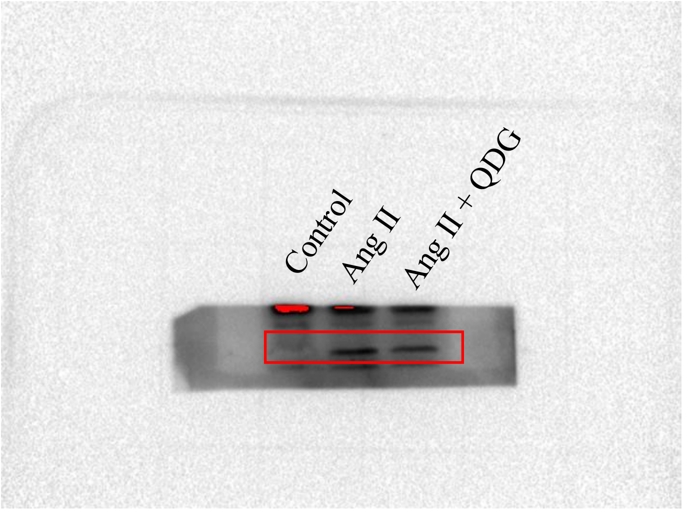

p53

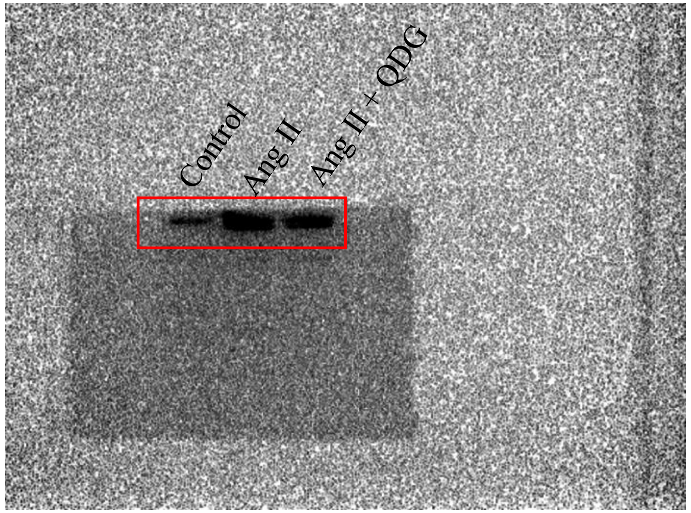
Control
Ang II
Ang II + QDG

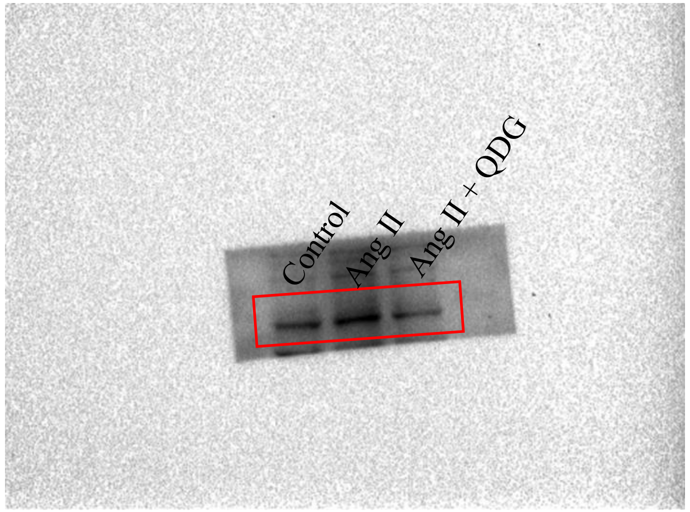
Control
Ang II
Ang II + QDG

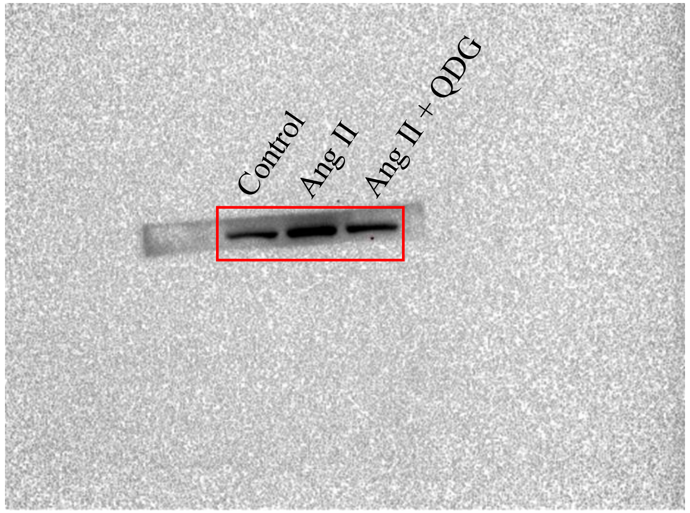
Control
Ang II
Ang II + QDG

GAPDH

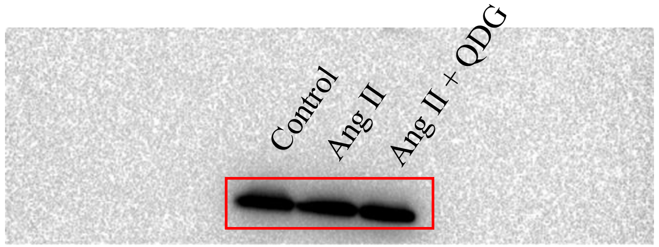
Control
Ang II
Ang II + QDG

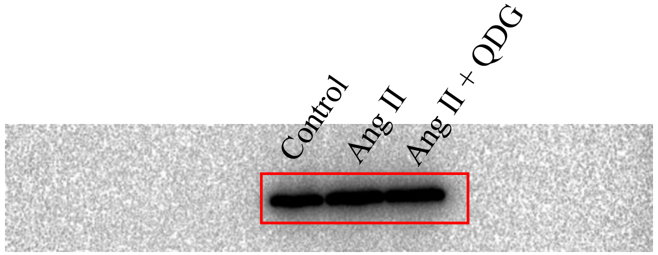
Control
Ang II
Ang II + QDG

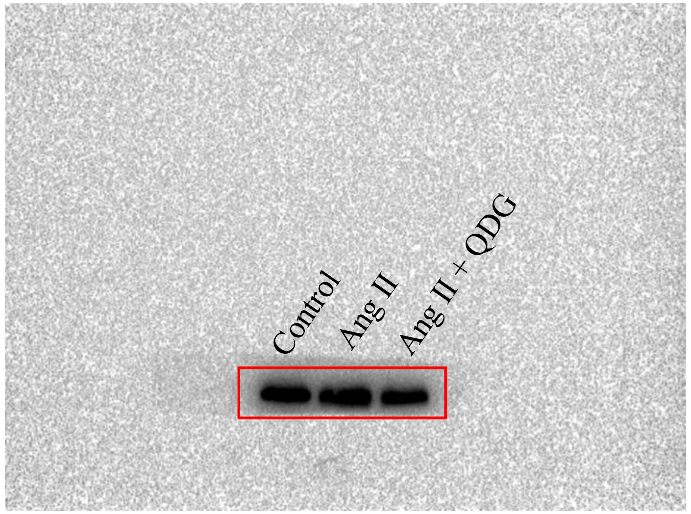
Control
Ang II
Ang II + QDG

Figure1-D

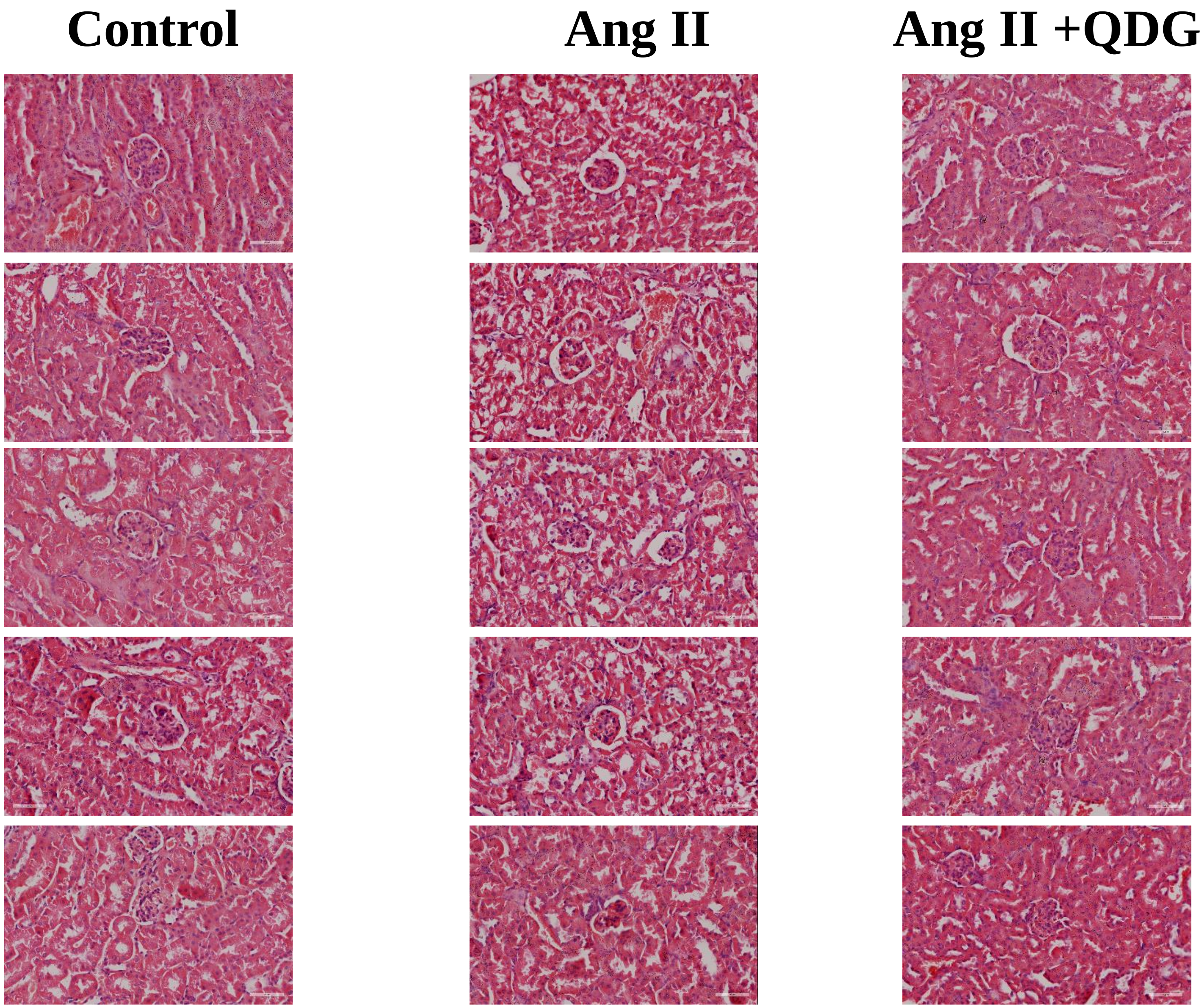

Figure5-A

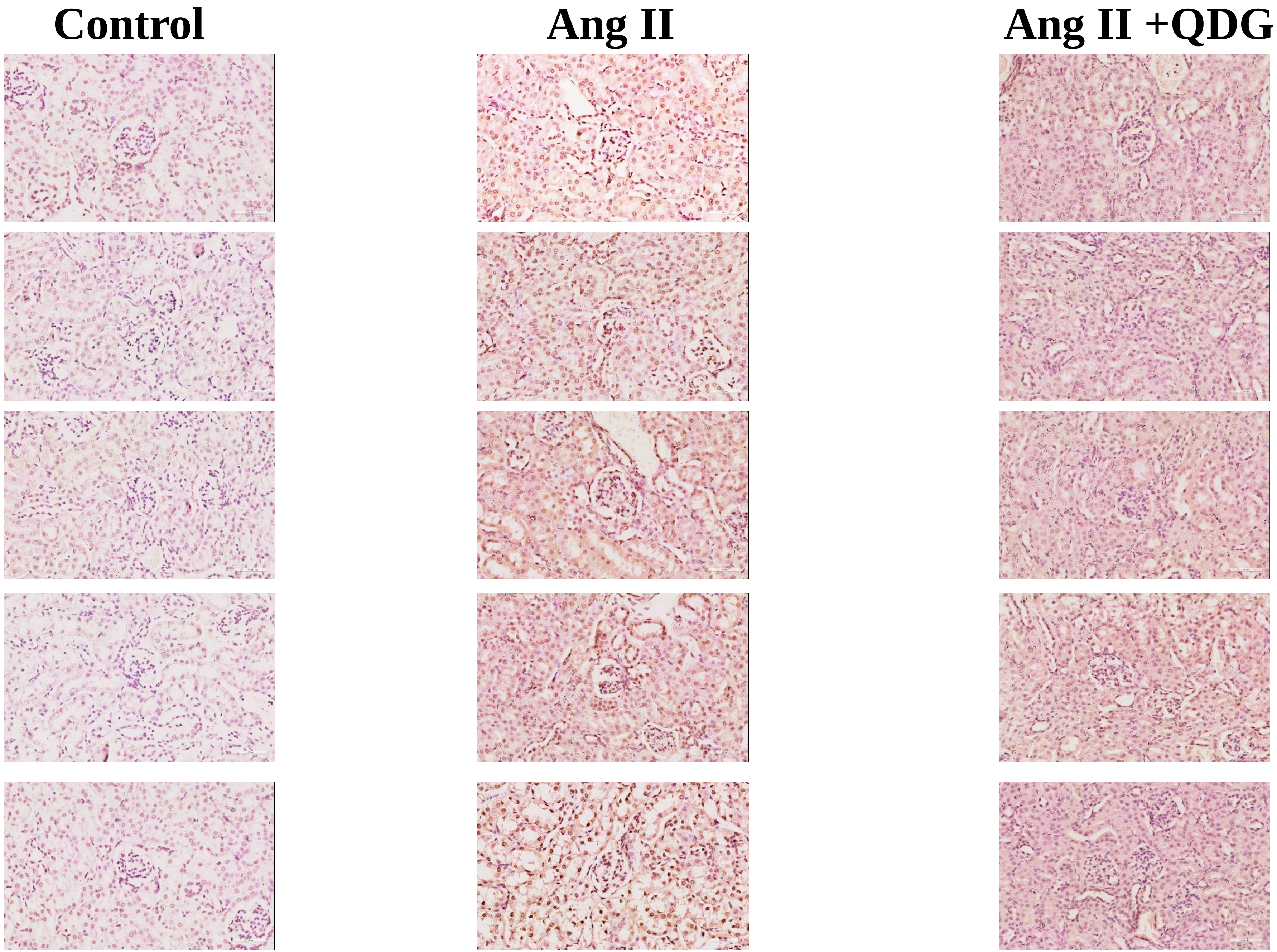

Ang II (1 µM)	-	+	+	+
QDG(µg/ml)	-	-	25	50

Figure5-E

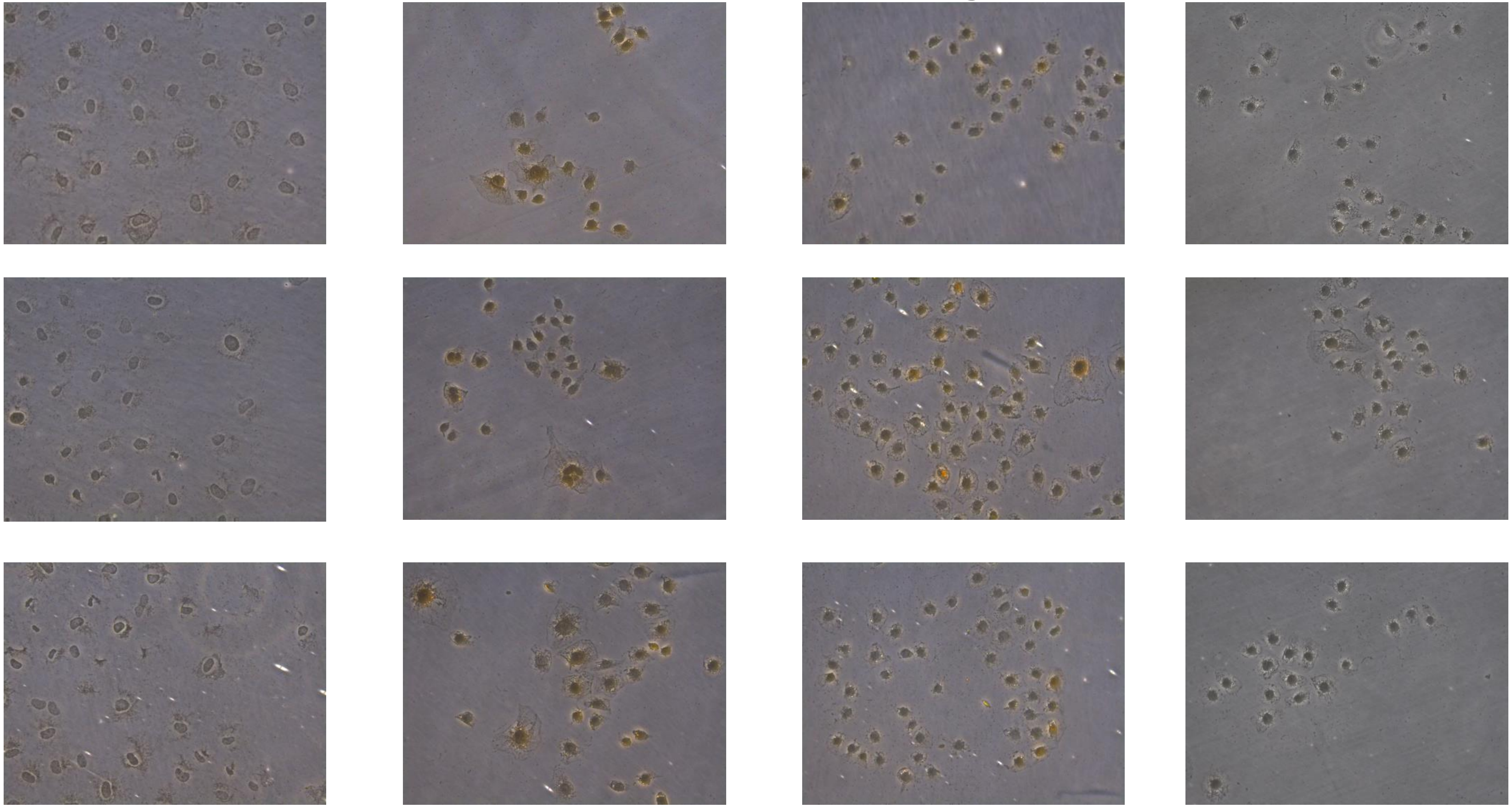

Supplement: Supplementary file 3 [file DataSheet1.PDF]
