# Supplementary material for: Qingda Granule Attenuates Angiotensin II-Induced Renal Apoptosis and Activation of the p53 Pathway
Source: Front Pharmacol. 2022 Feb 10;12:770863. doi: 10.3389/fphar.2021.770863 (PMC8867011; doi:10.3389/fphar.2021.770863)
Supplement: Supplementary file 6 [file Table6.DOCX]

**Control:**

**Ang II:**

**Ang II+QDG(25ug/ml)**

**Ang II+QDG(50ug/ml)**
